# Integrative single-cell RNA sequencing and bulk RNA sequencing reveals the characteristics of glutathione metabolism and protective role of GSTA4 gene in pancreatic cancer

**DOI:** 10.3389/fimmu.2025.1571431

**Published:** 2025-05-01

**Authors:** Xinya Jia, Qiang Zhang, Zhe Wang, Jianliang Cao, Anran Song, Chao Lan, Yuepeng Hu

**Affiliations:** ^1^ Department of Emergency Medicine, The First Affiliated Hospital of Zhengzhou University, Zhengzhou, China; ^2^ Department of Urology, Henan Provincial People’s Hospital, People’s Hospital of Zhengzhou University, Zhengzhou, China; ^3^ Department of Physical Diagnosis, The First Affiliated Hospital of Zhengzhou University, Zhengzhou, China

**Keywords:** single-cell RNA sequencing, bulk RNA sequencing, GSH metabolism, GSTA4 gene, pancreatic cancer

## Abstract

**Background:**

Recent studies have increasingly reported abnormal glutathione (GSH) metabolism within the tumor microenvironment across various solid tumors. However, the specific mechanisms underlying aberrant GSH metabolism in pancreatic cancer (PC) remain unclear. This study aims to investigate the prognostic significance of GSH metabolism-related genes in PC and to identify key molecular targets, thereby providing novel perspectives for targeted PC therapy.

**Methods:**

The GSH metabolism gene set was retrieved from the KEGG database. Utilizing single-cell transcriptomic data from the GSE205049 dataset, this study analyzed the variation in GSH metabolic signaling intensity across distinct cell types within the tumor microenvironment of PC. Additionally, transcriptomic data from multiple repositories, including TCGA, ICGC, and GEO, comprising a total of 930 patients with PC, were integrated to construct a prognostic molecular classifier related to GSH metabolism. Furthermore, the role of the key gene GSTA4 in PC was experimentally validated through a series of *in vitro* assays.

**Results:**

Significant differences in GSH metabolic signaling intensity were observed across various cell types in both normal pancreatic and PC tissues. A prognostic signature comprising six GSH metabolism-related genes (GSTA5, PGD, IDH2, GSTA4, GPX2, and GPX3) was established, wherein a high-risk score was associated with a poorer patient prognosis. Notably, GSTA4 expression was significantly reduced in PC tissues, and higher GSTA4 levels were linked to a favorable prognosis. *In vitro* functional analyses demonstrated that GSTA4 overexpression markedly inhibited PC cell proliferation and migration.

**Conclusion:**

The GSH metabolism-associated prognostic signature developed in this study effectively identifies high-risk patients with PC. As a prognostic protective factor, GSTA4 exhibits downregulated expression in PC tissues and suppresses tumor proliferation and migration, highlighting its potential as a therapeutic target.

## Introduction

Pancreatic cancer (PC), one of the most lethal gastrointestinal malignancies, has witnessed a more than twofold increase in incidence over the past two decades, imposing a substantial burden on global public health systems (1, 2). Despite significant advancements in PC treatment, including the introduction of innovative modalities such as immune checkpoint inhibitors and CAR T cell therapy, most cases are diagnosed at advanced stages, resulting in a poor overall prognosis with a 5-year survival rate of approximately 13% (3, 4). Consequently, identifying valuable molecular markers remains a critical objective in PC research, as their discovery could significantly enhance early diagnostic accuracy and facilitate precision medicine through individualized targeted therapy (5).

Emerging evidence indicates that metabolic reprogramming is a pivotal factor in the progression of PC. The persistent proliferation of tumor cells necessitates increased nutrient uptake and anabolic activity (6). This metabolic reprogramming primarily stems from intrinsic factors, including a range of metabolite-related genetic alterations. The PC tumor microenvironment is typically characterized by abnormal conditions, such as hypoxia and elevated lactic acid levels, which not only facilitate metabolic reprogramming but also induce adaptive metabolic shifts in tumor cells to ensure survival under hypoxic and acidic conditions (7). Furthermore, inflammatory mediators within the PC microenvironment play a pivotal role in tumor progression by interacting with various metabolic pathways, modulating the tumor microenvironment, and promoting the formation of a dense fibrotic matrix (8).

Disruptions in glutathione (GSH) metabolism are significantly associated with the development of various cancers. GSH is essential for maintaining protein homeostasis and mitigating oxidative stress within cells (9). The regulation of the cellular redox state is fundamental to both cancer initiation and progression. As the most critical intracellular antioxidant, GSH modulates tumor cell survival within the tumor microenvironment by scavenging excessive reactive oxygen species (ROS) generated from the endoplasmic reticulum (10). Moreover, GSH metabolism is intricately linked to cancer therapy and the emergence of drug resistance. Recent studies have established that resistance to multiple chemotherapeutic agents is closely associated with altered GSH metabolism. For instance, anticancer drugs such as cisplatin and paclitaxel exert their therapeutic effects by generating ROS, while elevated GSH levels neutralize these ROS, thereby diminishing drug cytotoxicity and fostering chemoresistance (11). Additionally, GSH has been implicated in mediating chemoresistance in PC through the ferroptosis pathway, characterized by the accumulation of lipid ROS (12, 13). Ferroptosis, driven by disrupted iron homeostasis, further underscores the importance of redox regulation in tumor progression (14). Notably, recent findings indicate that multiple GSH metabolism-related genes are upregulated in PC stem cells and that decreasing GSH levels can sensitize PC cells to gemcitabine (15). This study focuses on the expression patterns of GSH metabolism-related genes within the PC tumor microenvironment and investigates their mechanistic roles in PC progression, aiming to identify novel therapeutic targets.

Although single-cell sequencing has advanced our understanding of tumor metabolism, its application to GSH metabolism in PC remains limited. Despite the ability of single-cell sequencing technology to capture the gene expression landscape of tumor cells at a single-cell resolution, the regulation of GSH metabolism genes extends beyond tumor cells to encompass non-tumor cells within the complex PC microenvironment, which includes a diverse array of immune cells, fibroblasts, and endothelial cells. This study elucidates the impact of GSH metabolism-related genes on the PC tumor microenvironment at the single-cell level and identifies GSTA4 as a critical prognostic molecular marker. Additionally, the expression of GSTA4 in PC was validated through comprehensive molecular biology experiments, revealing its pivotal role in regulating apoptosis and migration of PC cells. These findings provide a theoretical foundation for the development of novel targeted therapeutic strategies.

## Materials and methods

### Single-cell sequencing and analysis

The GSE205049 dataset (16), comprising single-cell sequencing data from nine PC samples and nine normal pancreatic tissues, was obtained from the GEO database. Single-cell data were imported using the CreateSeuratObject function, applying the following quality control criteria: nCount_RNA ≥ 1000, nFeature_RNA ≥ 200, nFeature_RNA ≤ 6000, and percent.mt ≤ 20. Data normalization was performed using the SCTransform function. To evaluate GSH metabolism activity for each cell type, five single-cell gene set computational methods were employed: AUCell, UCell, Add, singscore, and ssgsea (17, 18). The summed scores from these methods, termed “Scoring,” provided a more comprehensive and stable representation of GSH metabolic activity compared to individual algorithms. The GSH gene set was sourced from the KEGG_GSH_METABOLISM pathway (http://www.gsea-msigdb.org/gsea/msigdb/cards/KEGG_GSH_METABOLISM).

### Bulk RNA sequencing analysis

Publicly available gene expression data and associated clinical information were collected from TCGA, GEO, ICGC, and ArrayExpress. To ensure data consistency, samples without survival information were excluded, and batch effects were mitigated using the ComBat method from the R package “SVA” (19). The integrated dataset included multiple cohorts: GSE57495 (20), GSE28735 (21), GSE62452 (22), MTAB-6134, TCGA, ICGC-CA, and ICGC-AU, comprising a total of 930 PC samples with complete clinical annotations.

Non-negative matrix factorization (NMF) (23) was applied to classify the PC samples based on GSH gene expression profiles. NMF efficiently simplifies high-dimensional data, enhancing the interpretation of gene set expression patterns. Using the NMF package, 930 patients with PC were stratified into two molecular subtypes, C1 and C2. Survival analysis was performed to determine prognostic differences between these subtypes. To quantify GSH metabolic activity and hallmark pathway activity, the GSVA package was utilized. Additionally, the Estimate package predicted immune and stromal scores for each subtype. The immune microenvironment was characterized using multiple prediction algorithms, including TIMER, CIBERSORT, and QUANTISEQ (24). To develop GSH metabolism-related prognostic markers, the LASSO-Cox regression model was constructed. Model performance was assessed via survival analysis and ROC curve evaluation. The sample grouping strategy was as follows: First, PC samples from TCGA, GEO, and ArrayExpress were merged. Subsequently, 50% of these samples were randomly selected as the training set, while the remaining 50% formed validation set 1. All PC samples from the TCGA, GEO, and ArrayExpress databases were designated as validation set 2, while samples from the ICGC platform constituted validation set 3. Gene weight prediction was performed using the random forest algorithm. The BEST platform was employed to explore the clinical significance of GSTA4. Comparative analysis between cancerous and adjacent normal tissues assessed GSTA4 expression, alongside evaluations of its correlation with tumor stage and grade. Based on GSTA4 expression levels, patients were categorized into high and low expression groups, and survival outcomes were compared to elucidate the clinical relevance of GSTA4.

### Cell culture

The normal human pancreatic ductal cell line (H6C7) was obtained from BeNa Culture Collection (BNCC), while the human PC cell lines (BxPc-3, PANC-1, CFPAC-1) were provided by Procell Life Science & Technology Co., Ltd. The culture medium for H6C7 and PANC-1 cells consisted of 45 mL DMEM and 5 mL FBS. BxPc-3 cells were maintained in a medium comprising 45 mL RPMI 1640 and 5 mL FBS. CFPAC-1 cells were cultured in a medium containing 45 mL IMDM and 5 mL FBS. All cell lines were incubated at 37°C with 5% CO_2_.

### RNA reverse-transcription and PCR experiment

Upon reaching full confluence, cells were digested with trypsin, followed by centrifugation at 1000 rpm for 5 minutes to collect the cell pellet. The supernatant was discarded, and the pellet was resuspended in 1 mL Trizol, mixed thoroughly, and allowed to stand for 5–10 minutes. Subsequently, 200 μL chloroform was added, and the mixture was centrifuged at 12,000 g for 15 minutes. The upper aqueous phase was carefully collected, mixed with 500 μL isopropanol, and centrifuged again at 12,000 g for 10 minutes. The supernatant was discarded, and the pellet was washed with 80% ethanol, followed by centrifugation at 7500 g for 5 minutes. After removing the supernatant, the pellet was air-dried and dissolved in an appropriate volume of RNase-free water for RNA concentration measurement.

RNA was reverse transcribed into cDNA using an All-in-One reverse transcription kit, following the manufacturer’s protocol. Quantitative real-time PCR (qRT-PCR) was performed using the SYBR Green method, adhering strictly to reagent preparation and machine operation guidelines.

### Transfection of the overexpression plasmid

The human GSTA4 overexpression plasmid and corresponding control plasmid were obtained from the gene supplier. Transfection was conducted using the Lipo3000 kit as follows: System 1 consisted of Opti-MEM™ medium (serum-free) mixed with Lipo3000; System 2 consisted of Opti-MEM™ medium (serum-free) combined with plasmid DNA and P3000™ reagent. After allowing the mixed System 2 solution to interact with System 1 for 10–15 minutes, the resulting complex was added to the cells.

### CCK8 experiment

Following transfection in 6-well plates, cells were digested with trypsin, and a defined number of PC cells were seeded into each well of a 96-well plate. After incubation at 37°C with 5% CO_2_ for 48-hour, a medium containing 10% CCK-8 was added to each well. After 1 hour of incubation, absorbance at 450 nm was measured using a microplate reader.

### Wound-healing experiment

For the wound-healing assay, transfected PC cells in the logarithmic growth phase were seeded in six-well plates at appropriate density and streaked using a 200 μL pipette tip. Images were captured at 0 and 48 hours post-scratch. During the migration period, cells were maintained in a low-serum medium to minimize proliferation interference.

## Results

### Enrichment score of GSH metabolism-related genes in scRNA-seq

**Figure 1:**
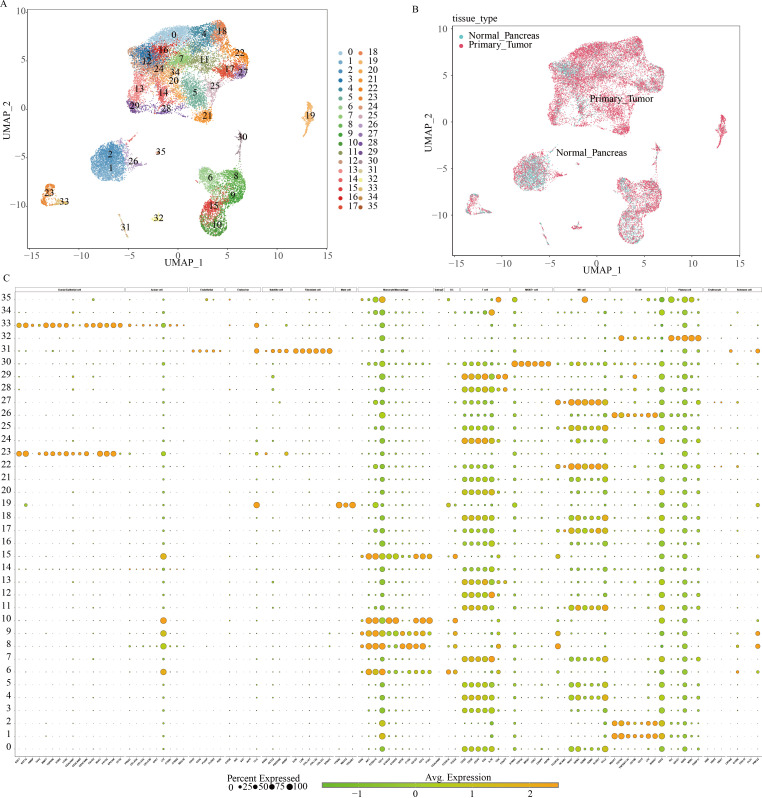
Classification of cell subpopulations based on scRNA data from pancreatic cancer. **(A)** UMAP plots visualizing distinct cell cluster classifications using varied color schemes and coding systems. **(B)** Differential cell distribution between normal pancreatic tissue and pancreatic cancer tissue of origin, represented by distinct colors within a single-cell UMAP landscape. **(C)** Marker gene expression profiles across multiple cell clusters.

Initially, nine paired tissue samples from PC and adjacent normal tissues were collected to assess the heterogeneity of GSH metabolism-related genes using single-cell sequencing data. Following the methodological criteria, the top 2000 hypervariable genes were identified and dimensionally reduced using principal component analysis (PCA). This process yielded 35 distinct cell clusters, whose distribution was visualized via Uniform Manifold Approximation and Projection (UMAP) (**Figure 1A**). The UMAP panel distinctly separates tumor tissue cells from normal pancreatic tissue cells (**Figure 1B**). Cell subsets were annotated based on known marker genes, and the annotation process was systematically documented. The bubble diagram (**Figure 1C**) illustrates the differential expression levels of marker genes across the 35 cell clusters, highlighting the heterogeneity among cell types.

**Figure 2:**
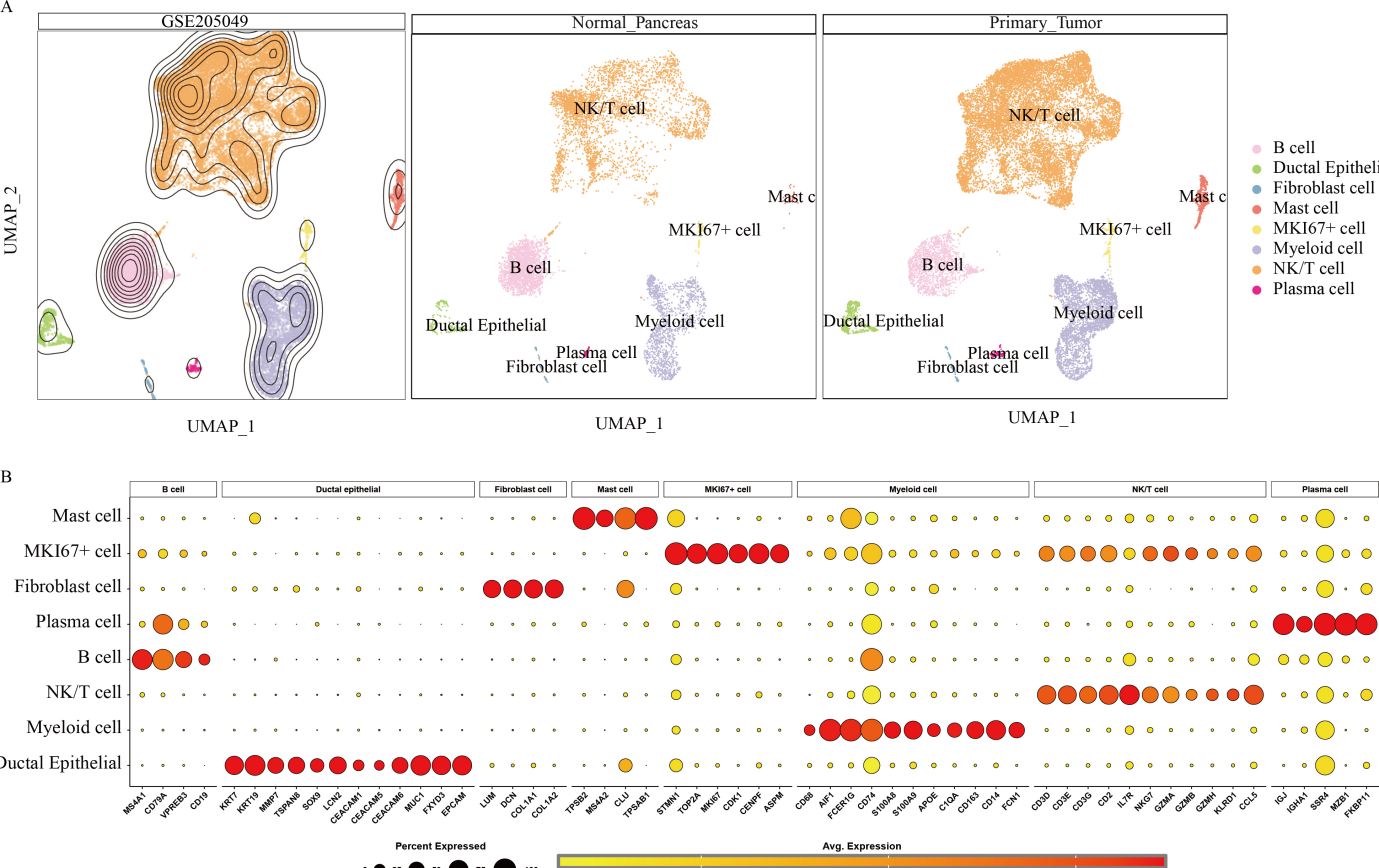
Annotation results for scRNA data from 9 pancreatic cancer tissues and 9 normal pancreatic tissues. **(A)** UMAP landscape illustrating the distribution of various cell types within normal pancreatic tissue and pancreatic cancer tissue. **(B)** Marker gene expression profiles corresponding to cell type identification during annotation.

To further investigate the heterogeneity within the PC tumor microenvironment, the cellular composition of primary tumor and normal pancreatic samples was analyzed. Cell populations were represented with distinct colors and markers, including B cells (CD79A), ductal epithelial cells (EPCAM), fibroblasts (LCM), mast cells (TPSAB1), MKI67+ proliferative cells (MKI67), myeloid cells (CD74), NK/T cells (CD3D, CD3E, GZMA), and plasma cells (IGJ) (**Figure 2A**). **Figure 2B** displays the relative expression levels of key marker genes within each cell subset.

**Figure 3:**
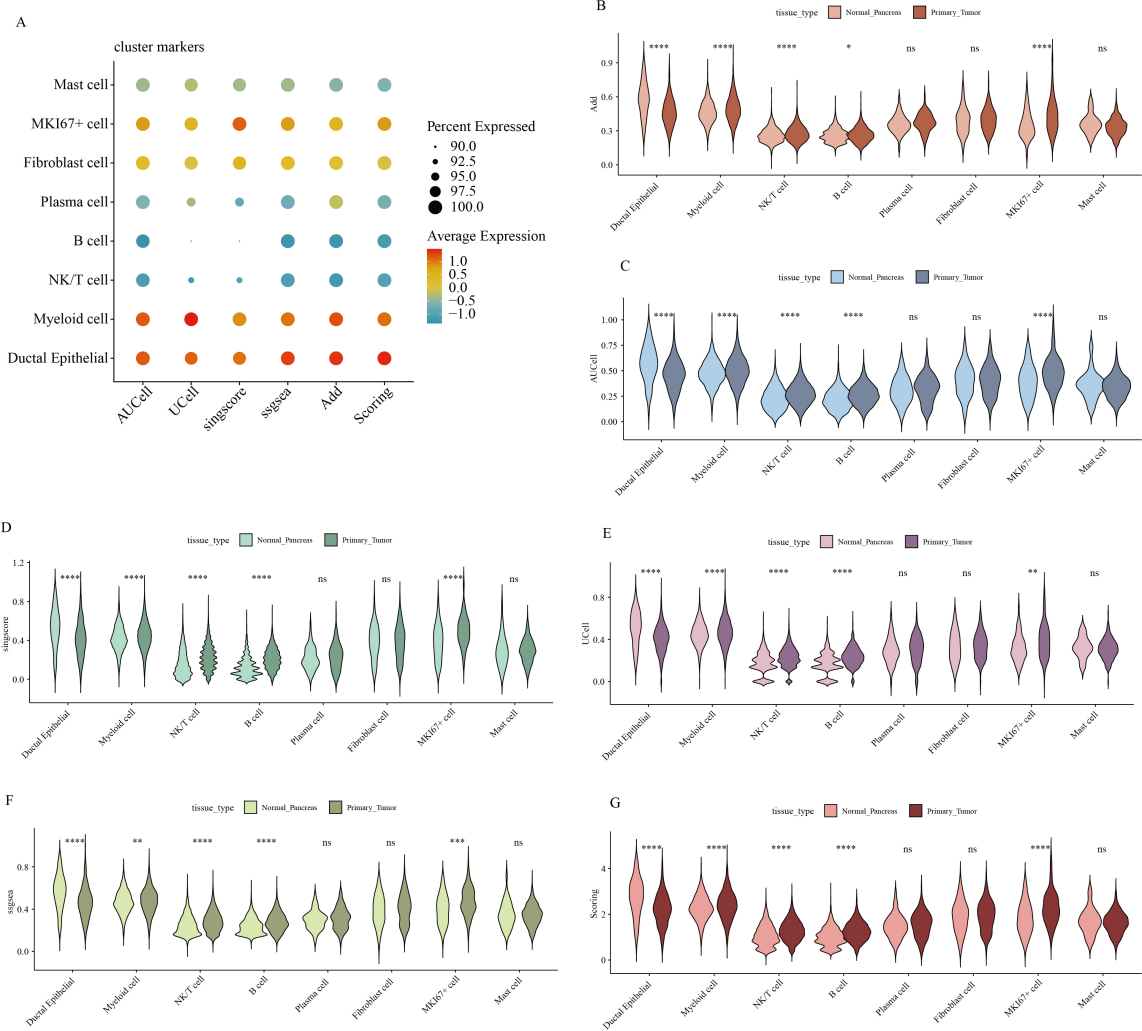
Gene expression scores associated with GSH metabolism in pancreatic cancer. **(A)** Comparison of gene expression scores related to GSH metabolism across different cell types, evaluated using six classical algorithms. **(B-G)** Comparative analysis of GSH metabolic signal scores across cell types between pancreatic and pancreatic cancer tissues. * indicates p <0.05; ** indicates p <0.01; *** indicates p <0.001; **** indicates p <0.0001.

Hypervariable genes were identified for each cell type, followed by correlation analysis of cell annotations. The analysis identified eight cell types: B cells (MS4A1, CD79A, VPREB3, CD19); ductal epithelial cells (KRT7, KRT19, MMP7, TSPAN8, SOX9, LCN2, CEACAM1, CEACAM5, CEACAM6, MUC1, FXYD3, EPCAM); fibroblasts (LUM, DCN, COL1A1, COL1A2); mast cells (TPSB2, MS4A2, CLU, TPSAB1); MKI67+ cells (STMN1, TOP2A, MKI67, CDK1, CENPF, ASPM); myeloid cells (CD64, CD68, AIF1, FCER1G, CD74, S100A8, S100A9, APOE, C1QA, CD163, CD14, FCN1); NK/T cells (CD3D, CD3E, CD3G, CD2, IL7R, NKG7, GZMA, GZMB, GZMH, KLRD1, CCL5); and plasma cells (IGJ, IGHA1, SSR4, MZB1, FKBP11) To quantify the expression of 50 GSH metabolism-related genes (**Table 1**) across different cell types, gene set scoring was performed using six algorithms: AUCell, UCell, singscore, ssgsea, Add, and Scoring. All six methods consistently indicated that GSH metabolism was most active in myeloid cells and ductal epithelial cells, followed by MKI67+ cells and fibroblasts (**Figure 3A**). Subsequently, GSH metabolic signal intensity was compared between PC tumor tissues and normal pancreatic tissues using the six algorithms, presented as violin plots (**Figures 3B–G**). The analyses revealed that GSH metabolism-related genes exhibited significantly higher expression in tumor tissues, particularly within myeloid cells, NK/T cells, B cells, and MKI67+ cells, compared to normal pancreatic samples. In contrast, ductal epithelial cells demonstrated relatively low GSH metabolic gene expression. To further validate the spatial distribution of GSH metabolic activity, GSH signal scores were mapped onto UMAP representations for both normal pancreatic and PC tumor tissues, confirming the differential activity levels of GSH metabolism across various cell types (**Supplementary Figures 1**, **2**).

**Table 1:** 50 genes obtained from KEGG_GLUTATHIONE_METABOLISM pathway.

SRM	PGD	GSTM1	GSTM5	GSTK1	IDH1
GGT1	GSTO1	GPX7	GSTA1	GPX5	OPLAH
GSTP1	GSTA5	GSTA4	GSTA2	TXNDC12	GCLM
GSTT2	MGST2	GPX6	GSR	GPX1	GGT6
GSTT1	LAP3	GSTM4	GSS	GPX2	ANPEP
GSTZ1	MGST1	GGCT	RRM1	GPX3	
RRM2B	MGST3	GSTM3	RRM2	IDH2	
SMS	GSTA3	GSTM2	GCLC	GPX4	
G6PD	ODC1	GGT5	GSTO2	GGT7	

### NMF cluster analysis of GSH metabolism-related genes

**Figure 4:**
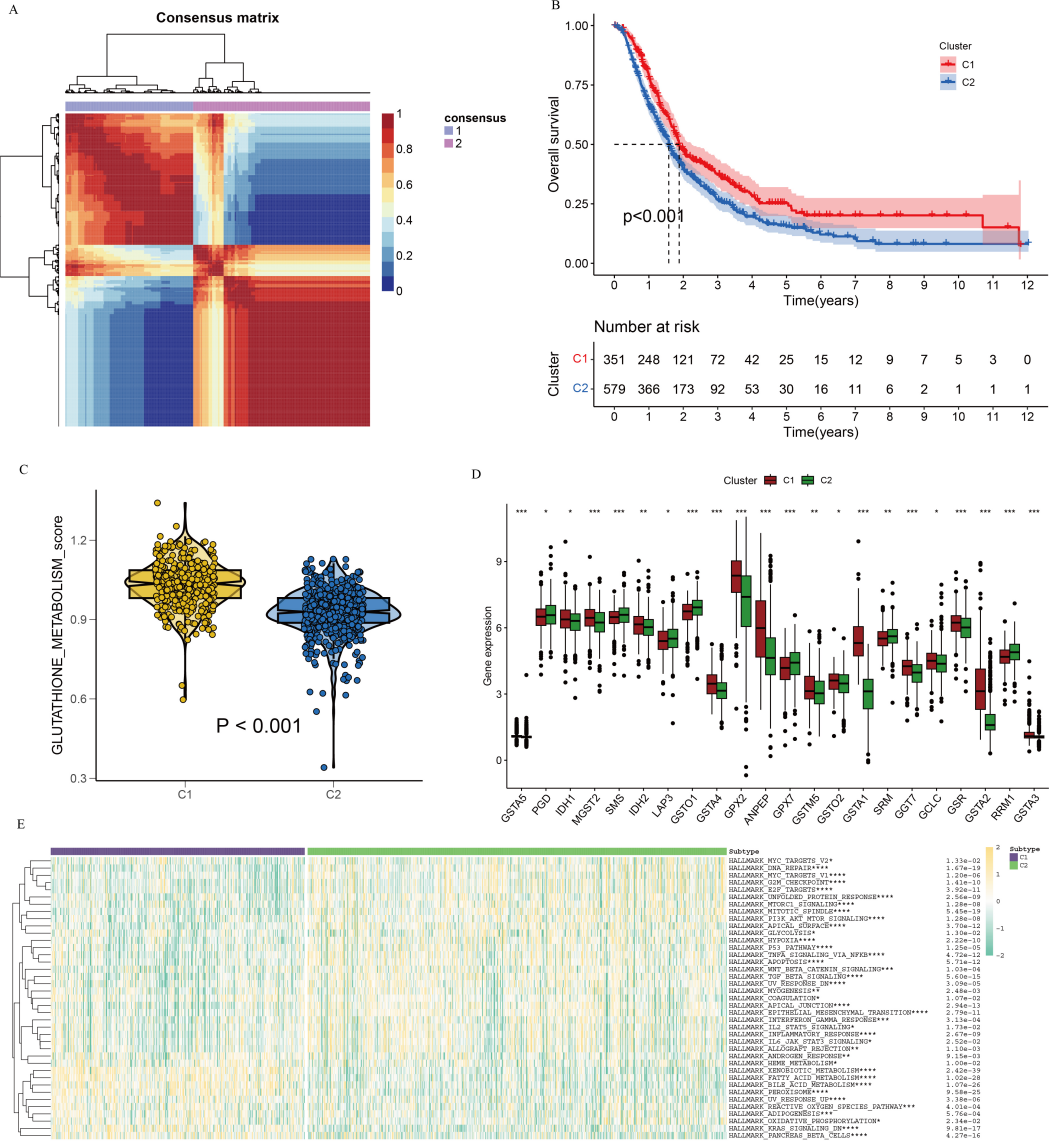
NMF clustering of bulk-RNA data from 930 pancreatic cancer patients, delineating two distinct subgroups. **(A)** Consensus map illustrating NMF cluster distribution. **(B)** Kaplan-Meier curve depicting cluster-specific survival differences based on GSH metabolism-related genes. **(C)** Violin plots illustrating expression differences of GSH metabolism-related genes between the two clusters, derived using the GSVA algorithm. **(D)** Differential gene expression related to GSH metabolism between clusters. **(E)** Heatmap depicting pathway enrichment for differentially expressed genes in the two clusters. * indicates p <0.05; ** indicates p <0.01; *** indicates p <0.001; **** indicates p <0.0001.

To evaluate the prognostic significance of GSH metabolic signaling in PC, comprehensive transcriptomic analyses integrating bulk RNA-seq data and prognostic information from patients with PC were were performed across multiple datasets. Initially, the NMF algorithm was applied to cluster 930 patients with PC into molecular subtypes. Various performance metrics, including contour coefficient, dispersion, silhouette score, and sparsity, were calculated to determine the optimal rank value, ensuring clustering validity and stability (**Supplementary Figure 3**). Based on the integration of multiple evaluation criteria, two clusters (C1 and C2) were selected as the optimal solution. The expression profiles of GSH metabolism-related genes between the two clusters revealed significantly higher expression in the C1 cluster compared to the C2 cluster (**Figure 4A**). Prognostic analysis indicated that patients in the C2 cluster exhibited worse overall survival compared to those in the C1 cluster, suggesting that elevated GSH metabolism gene expression is associated with poor prognosis in PC (**Figures 4B, C**). The heightened GSH metabolism signal activity was further validated within the C1 cluster.

To investigate the interaction between GSH metabolic signaling and immune system dynamics within the tumor microenvironment, an immune checkpoint analysis targeting differentially expressed genes in relation to GSH metabolic signaling was performed. Results indicated significant variations in the expression of most GSH metabolism-related genes between the two clusters (**Figure 4D**). Differential pathway analysis between C1 and C2 clusters, visualized via heatmap, identified several distinct metabolism-related signaling pathways, including xenobiotic metabolism, fatty acid metabolism, and bile acid metabolism (**Figure 4E**).

**Figure 5:**
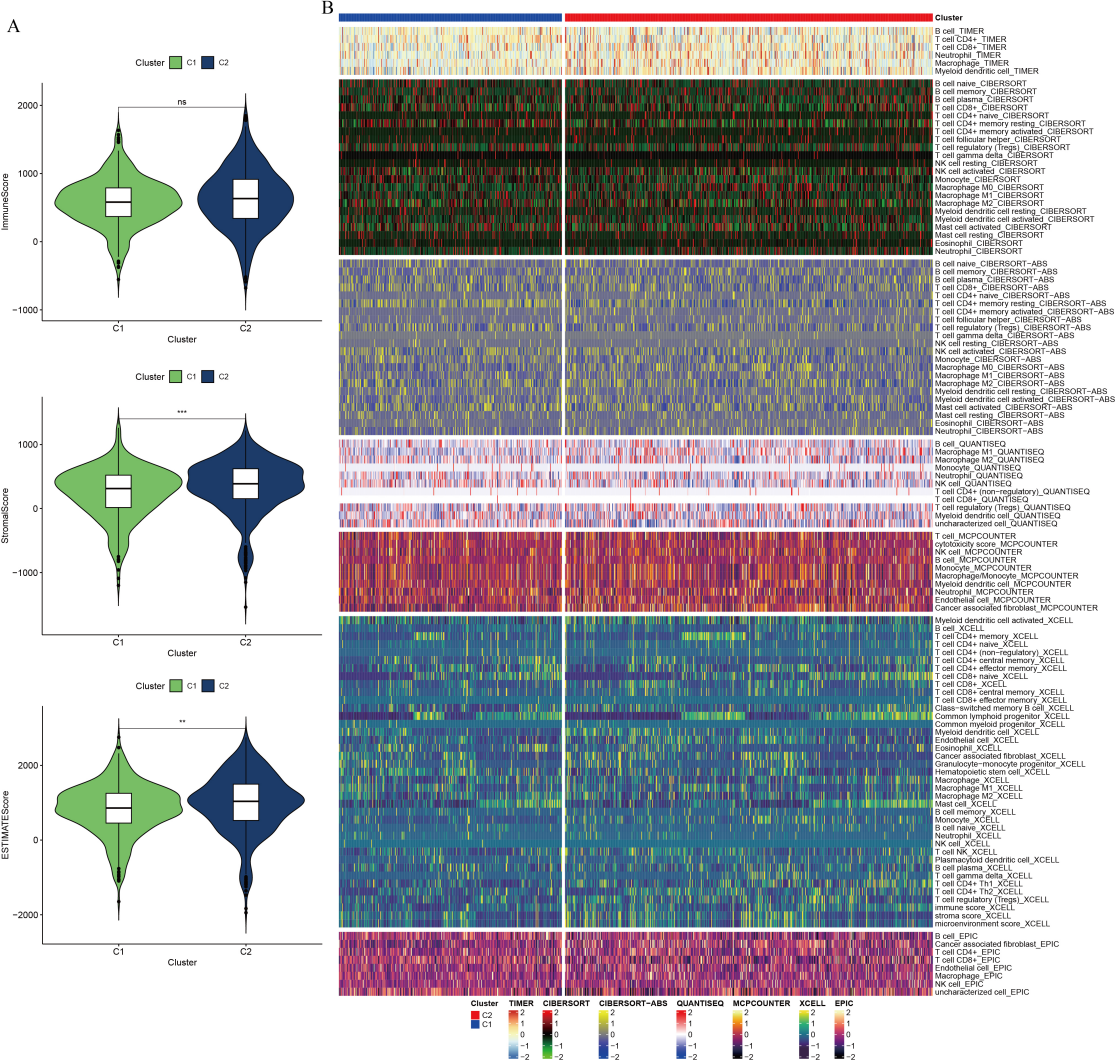
Differential analysis of immune composition in the tumor microenvironment of pancreatic cancer, based on NMF clustering. **(A)** Violin plot comparing ESTIMATE score, immune component score, and stromal component score between clusters. **(B)** Comparative analysis of immune cell infiltration between clusters using seven computational algorithms. ** indicates p <0.01; *** indicates p <0.001.

To assess the immune landscape within the PC tumor microenvironment, immunity, stromal, and tumor scores were calculated using the ESTIMATE algorithm. The C2 cluster demonstrated higher ESTIMATE and stromal scores compared to the C1 cluster, although no significant difference in immune scores was observed between the groups (**Figure 5A**). To further dissect the immune cell composition within the PC tumor microenvironment, seven computational algorithms were employed: TIMER, CIBERSORT, CIBERSORT-ABS, QUANTISEQ, MCPcounter, xCell, and EPIC (**Figure 5B**). Cross-validation using these algorithms revealed distinct immune cell infiltration patterns between groups with high and low GSH metabolic signaling. Notably, increased infiltration of immune cells such as CD4+ T cells, CD8+ T cells, B cells, and neutrophils was observed in the high GSH metabolism expression group, indicating an immunosuppressive microenvironment in PC characterized by elevated GSH metabolism gene expression. Subsequently, immune checkpoint gene analysis between the C1 and C2 clusters identified significant differential expression of several checkpoint-related genes, suggesting that variations in GSH metabolic signaling may influence immune escape mechanisms (**Supplementary Figure 4**). To evaluate the relationship between GSH metabolic signaling activity and therapeutic response, a drug sensitivity analysis was conducted. Patients with lower expression of GSH metabolism-related genes exhibited higher sensitivity to specific targeted therapies, including KRAS (G12C) inhibitors, Lapatinib, Sorafenib, and Gefitinib (**Supplementary Figure 5**).

### Construction and validation of a prognostic model related to GSH metabolism

**Figure 6:**
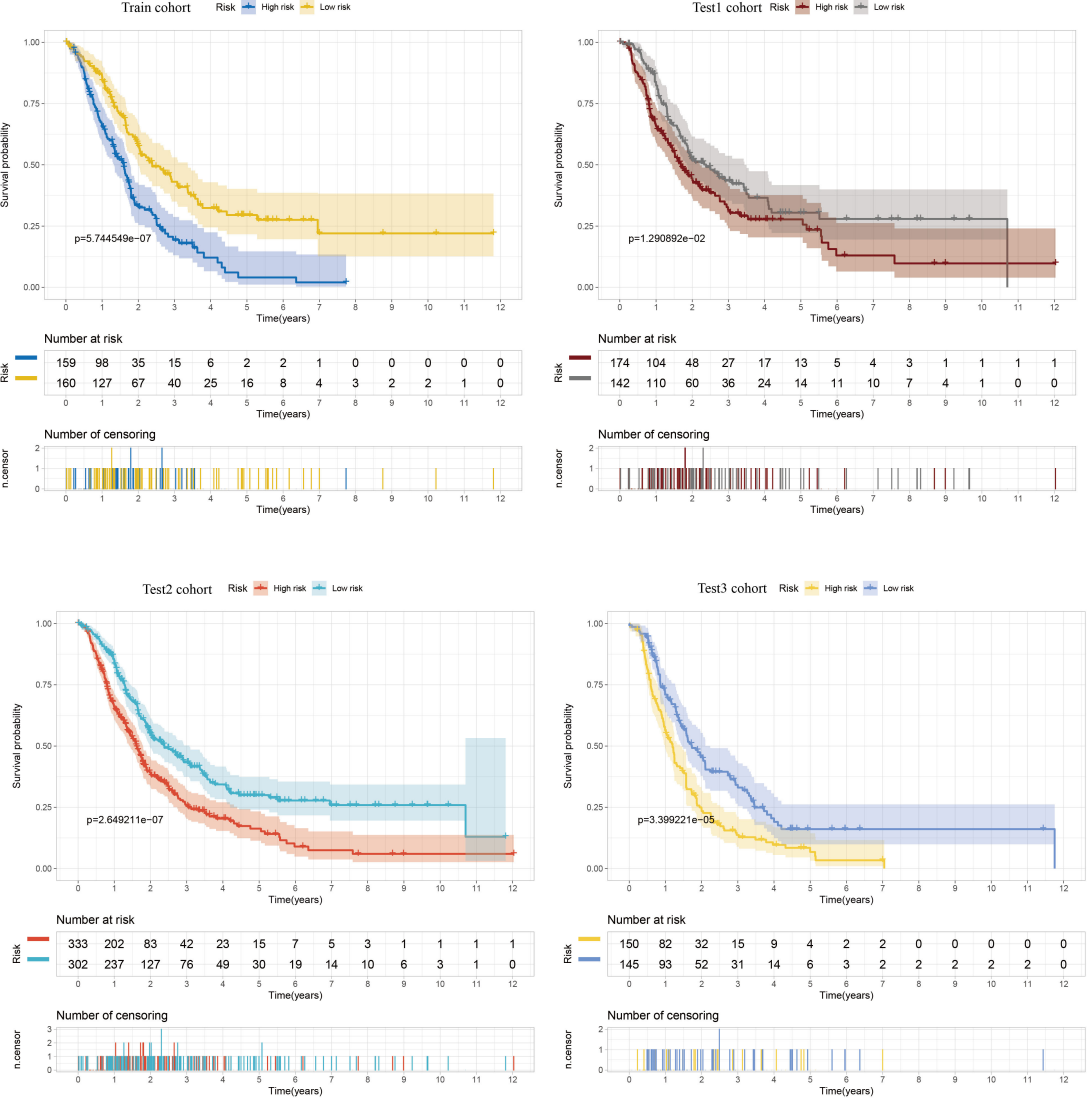
Kaplan-Meier curves comparing high and low risk scores between training and three validation cohorts.

**Figure 7:**
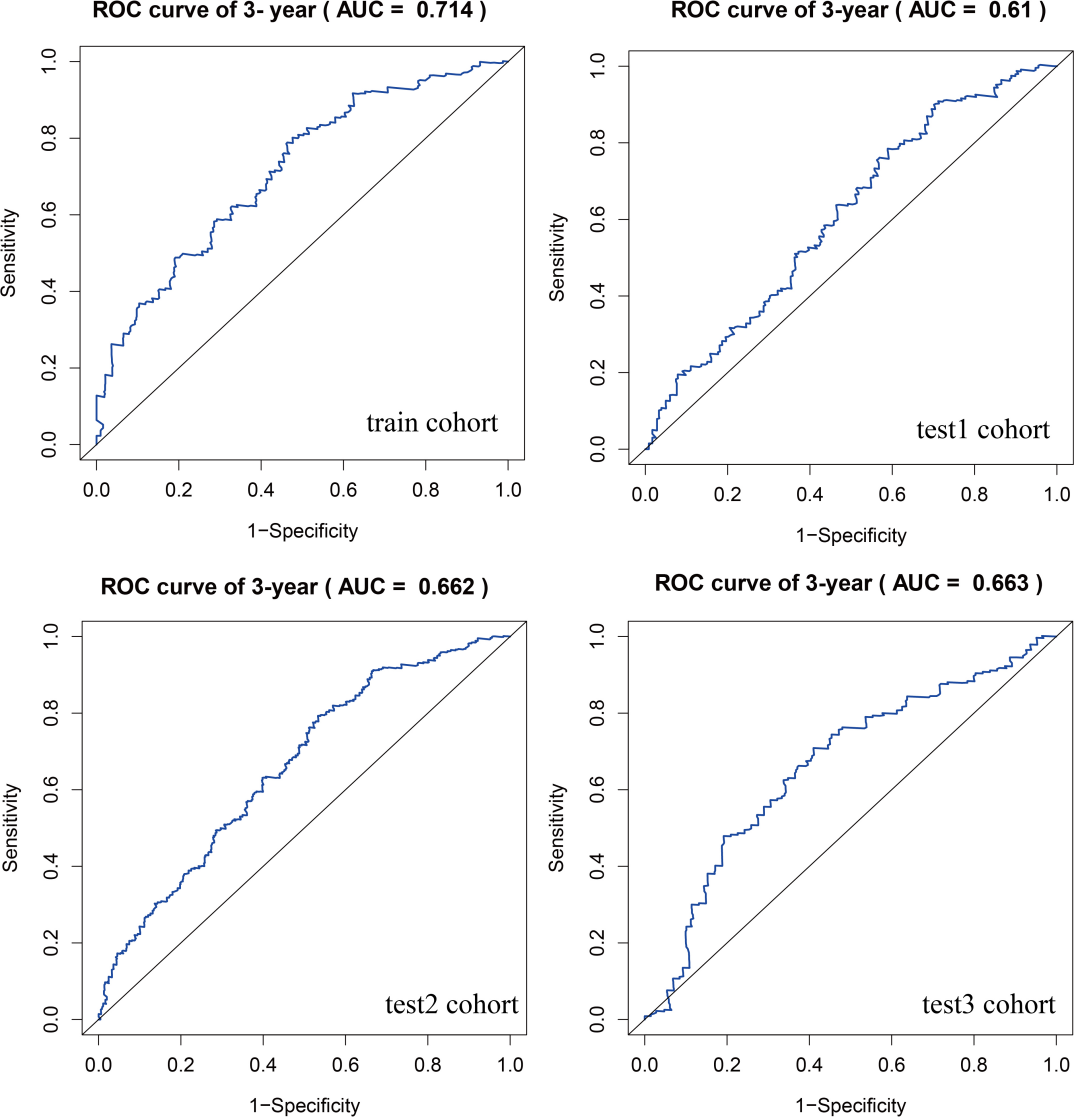
ROC curves comparing high and low risk scores between training and three validation cohorts.

To identify key genes associated with GSH metabolism in PC, the classical LASSO regression model was employed to construct a prognostic correlation model. The process of model construction, including the introduction of regularization to constrain parameters and enable variable selection, is detailed in **Supplementary Figure 6**. Following this approach, six key GSH metabolism-related genes were selected: GSTA5, PGD, IDH2, GSTA4, GPX2, and GPX3. The expression levels of the six model genes across different cohorts, stratified by high and low-risk groups, are presented in **Supplementary Figure 7**. For model validation, 930 patients with clinical prognostic data were partitioned into training and three independent validation sets. Survival curves were generated for the prognostic risk model across these cohorts, consistently demonstrating that patients classified in the high-risk group exhibited poorer outcomes across both training and validation sets, thereby confirming the model’s robust prognostic accuracy and stability (**Figure 6**). To further assess the predictive performance of the model, 3-year Receiver Operating Characteristic (ROC) curves were generated for each cohort, with Area Under the Curve (AUC) values calculated accordingly (**Figure 7**). The 3-year AUC for the training cohort was 0.714, while the AUC values in the three validation cohorts consistently exceeded 0.6. These results underscore the model’s efficacy in differentiating between high-risk and low-risk patients with PC.

### Molecular biological validation of GSTA4 in pancreatic cancer

**Figure 8:**
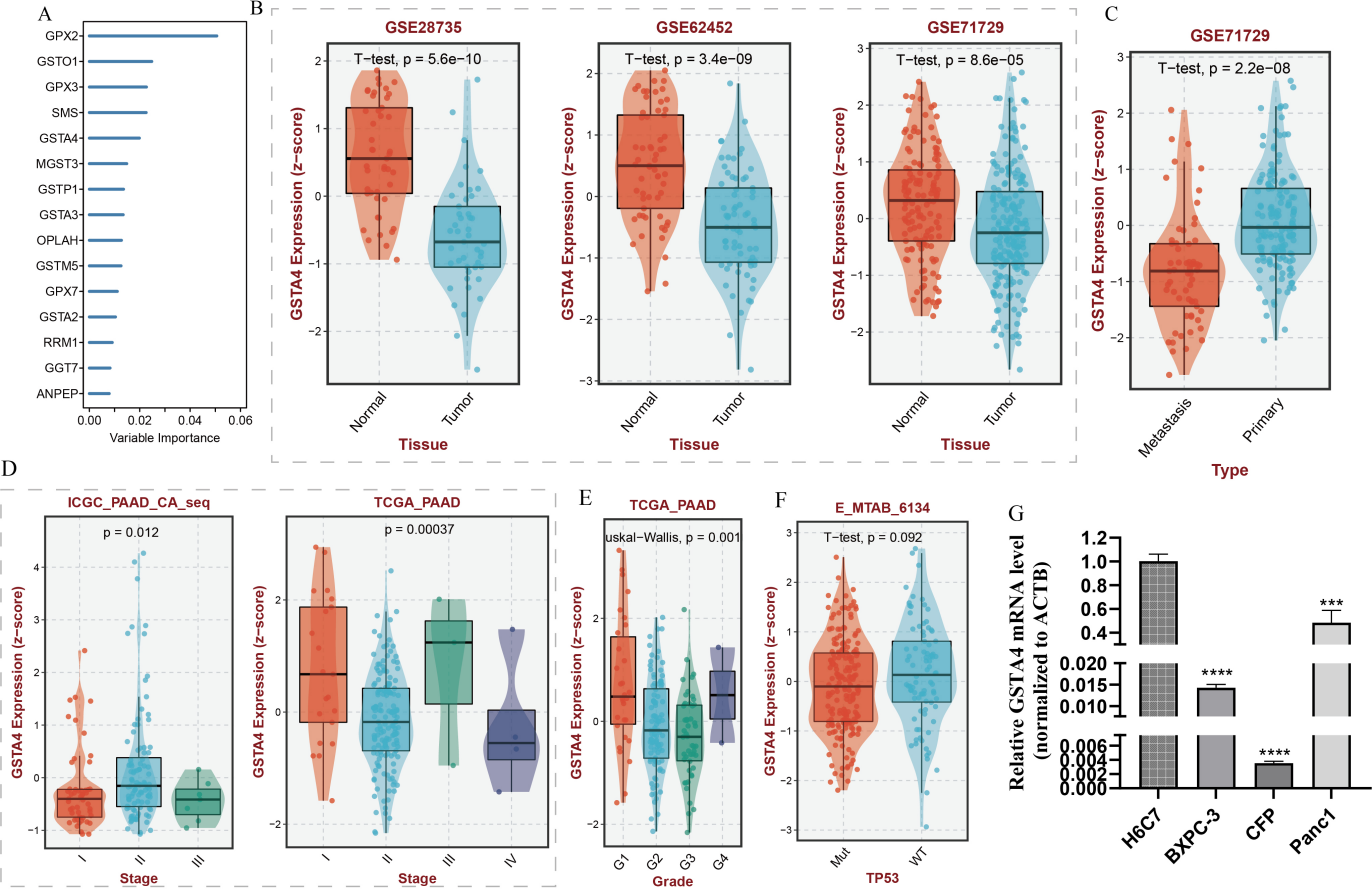
Random forest analysis of GSH metabolism-associated genes and correlation analysis between GSTA4 expression and clinical features in pancreatic cancer. **(A)** Random forest analysis identifying key GSH metabolism-related genes. **(B)** Differential GSTA4 expression between normal pancreatic tissue and pancreatic cancer tissue across three cohorts. **(C)** Comparison of GSTA4 expression between primary and metastatic pancreatic cancer. **(D)** Correlation between GSTA4 expression and tumor staging within the pancreatic cancer cohort. **(E)** Correlation between GSTA4 expression and tumor grading within the pancreatic cancer cohort. **(F)** Correlation between GSTA4 expression and TP53 mutation within the pancreatic cancer cohort. **(G)** PCR-based assessment of GSTA4 expression in the normal pancreatic cell line H6C7 and three pancreatic cancer cell lines (BXPC-3, CFP, Panc-1). *** indicates p <0.001; **** indicates p <0.0001.

**Figure 9:**
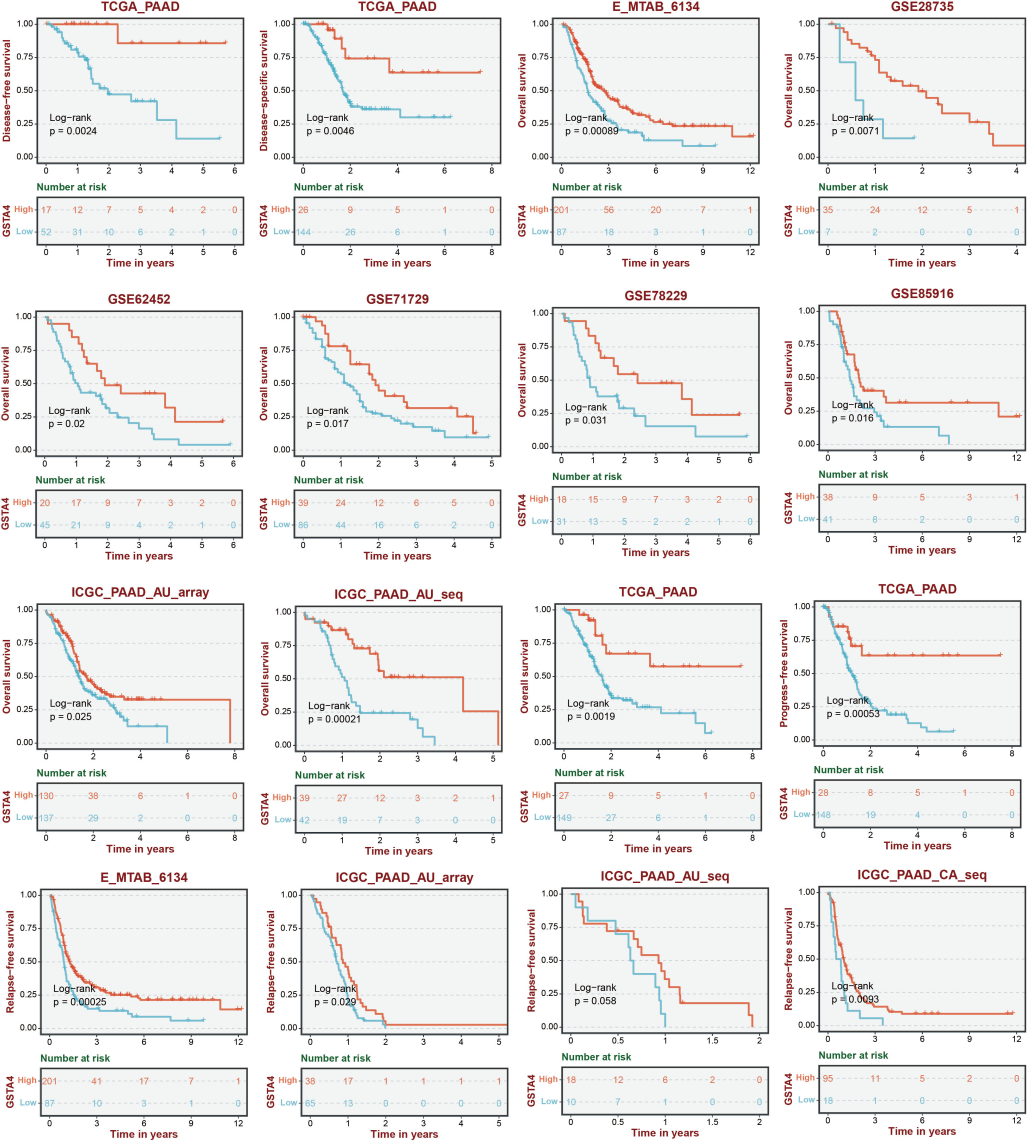
Survival analysis stratified by GSTA4 expression across various pancreatic cancer cohorts.

A random forest analysis was performed to investigate the significance of GSH metabolism in PC, and the results were visualized (**Figure 8A**). Among the identified genes, GPX2, GSTO1, GPX3, SMS, and GSTA4 demonstrated high weights in the PC context. Upon intersecting these results with the LASSO screen and conducting a literature review, it was noted that the mechanistic understanding of GSTA4 in PC remains limited. Therefore, a more detailed analysis was conducted to explore the correlation between GSTA4 and clinical characteristics in patients with PC. Analysis of the GSE28735, GSE62452, and GSE71729 datasets revealed significantly lower GSTA4 expression in PC tumor tissues compared to normal pancreatic tissues (**Figure 8B**). Additionally, metastatic PC tissues exhibited reduced GSTA4 expression relative to primary PC tissues (**Figure 8C**). Further investigations demonstrated an association between GSTA4 expression levels and tumor stage, tumor grade, and TP53 gene mutation status (**Figures 8D–F**). To further elucidate the prognostic relevance of GSTA4 in PC, survival analyses were performed across multiple cohorts of patients with PC, including disease-free survival, disease-specific survival, overall survival, progression-free survival, and relapse-free survival. These analyses encompassed the following cohorts: TCGA-PAAD, E-MTAB-6134, GSE28735, GSE62452, GSE71729, GSE78229, GSE85916, and ICGC-PAAD. Patients were stratified into high and low GSTA4 expression groups, and prognosis differences were assessed within each cohort. The results consistently indicated that patients with high GSTA4 expression had significantly better prognoses compared to those with low expression, identifying GSTA4 as a potential prognostic protective factor in PC (**Figure 9**).

**Figure 10:**
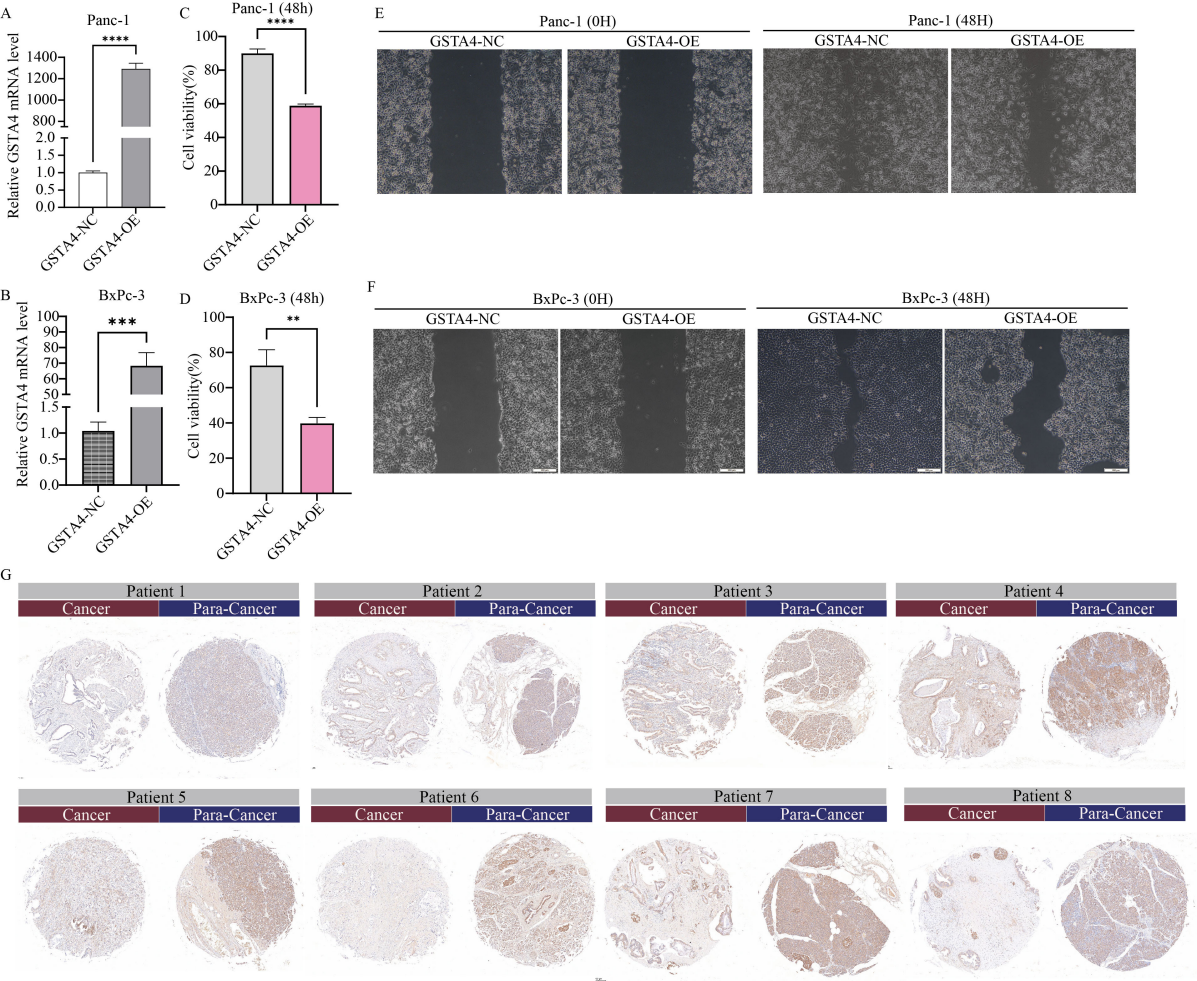
Functional characterization of GSTA4 in pancreatic cancer cells and immunohistochemical validation in clinical specimens. **(A, B)** PCR analysis demonstrated a significant upregulation of GSTA4 expression in overexpressing cell lines Panc-1 and BxPC-3. **(C, D)** CCK-8 assays revealed that GSTA4 overexpression markedly inhibited the proliferative capacity of Panc-1 and BxPC-3 pancreatic cancer cells *in vitro* at 48 hours. **(E, F)** Wound-healing assays indicated a significantly reduced migration rate in GSTA4-overexpressing cells after 48 hours. **(G)** Immunohistochemical analysis using tissue microarrays revealed differential GSTA4 expression patterns between pancreatic cancer tissues and adjacent normal tissues. ** indicates p <0.01; *** indicates p <0.001; **** indicates p <0.0001.

To experimentally validate the bioinformatics predictions, GSTA4 expression was examined in PC cell lines. PCR analysis demonstrated that GSTA4 expression was markedly lower in PC cell lines (BXPC-3, CFPAC-1, Panc1) compared to the normal pancreatic cell line H6C7 (**Figure 8G**). To investigate the functional role of GSTA4, overexpression models were established in BXPC-3 and Panc1 cell lines. Successful GSTA4 overexpression was confirmed in the transfected cell lines (**Figures 10A, B**). Functional assays revealed that GSTA4 overexpression significantly inhibited cell proliferation, as evidenced by CCK8 assays conducted at 48-hour, which showed reduced proliferation rates in GSTA4-overexpressing BXPC-3 and Panc1 cell lines (**Figures 10C, D**). Moreover, the wound-healing assay demonstrated a pronounced reduction in cell migration at the 48-hour mark in the GSTA4-overexpressing group compared to controls (**Figures 10E, F**). The immunohistochemistry experiment once again confirmed that GSTA4 exhibits an abnormally low expression characteristic in pancreatic cancer (**Figure 10G**).

## Discussion

Recent studies have increasingly highlighted the abnormal metabolic characteristics of the tumor microenvironment (25, 26), positioning metabolic targeting as a promising avenue in anticancer immunotherapy (27). As the understanding of GSH deepens, it has become evident that GSH not only maintains cellular redox homeostasis but also plays a pivotal regulatory role within the tumor microenvironment (28). Key features of the tumor microenvironment, such as hypoxia and acidosis, significantly influence the redox state of cells, and accumulating evidence indicates that such conditions are frequently associated with elevated GSH concentrations (29, 30). Emerging data suggest that tumor cells adapt to hypoxic and acidic environments by upregulating GSH-dependent antioxidant enzymes, including GSH peroxidase and GSH reductase, alongside increasing intracellular GSH levels (31). In response to excessive ROS production, the cellular antioxidant system is activated, with GSH functioning as a key antioxidant. This understanding has led to the hypothesis that inhibiting GSH synthesis or utilization could serve as a novel therapeutic strategy for cancer. Furthermore, GSH depletion within the tumor microenvironment has been linked to ferroptosis, a form of iron-dependent cell death (32). However, the mechanistic insights into abnormal GSH metabolism within the PC tumor microenvironment remain limited. Notably, Cai et al. demonstrated that inhibiting GSH metabolism could decelerate PC progression, though the molecular mechanisms underlying this effect remain unexplored (33). The present study systematically investigated the genes involved in GSH metabolism in PC, utilizing single-cell and transcriptomic data to identify key molecular markers associated with GSH metabolic dysregulation. The biological functions of these markers were experimentally validated using a series of molecular biology techniques.

The tumor microenvironment of solid tumors encompasses not only tumor cells but also a diverse array of non-tumor cell types, including myeloid cells, natural killer (NK) cells, and B cells, alongside numerous stromal components, chemokines, cytokines, and metabolites. These elements collectively form the complex network characteristic of the tumor microenvironment (34). Analysis of GSH metabolic signaling activity using single-cell data from normal pancreatic tissues and PC tissues revealed significant differences in signal intensity across various cell types, particularly ductal epithelial cells, myeloid cells, NK/T cells, and B cells.

The carcinogenesis of pancreatic ductal epithelial cells is intrinsically linked to the development of PC. During epithelial-stromal transformation (EMT), pancreatic ductal epithelial cells lose epithelial characteristics, including tight junctions and polarity, and acquire stromal traits, a process pivotal for cancer cell infiltration and metastasis (35, 36). Studies by Kim et al. have demonstrated that most EMT-associated proteins are involved in gemcitabine-resistant and sensitive GSH and cysteine/methionine metabolism within gemcitabine-resistant human pancreatic adenocarcinoma cell lines (37). GSH peroxidase-1 (GPX1), a key antioxidant enzyme, participates in GSH metabolism (38). GPX1 silencing induces ROS-mediated activation of the Akt/GSK3β/Snail signaling pathway, thereby promoting both EMT and the development of chemoresistance in PC (39). Additionally, oncogenic RAS activation in PC augments NADPH-oxidase activity, resulting in increased ROS production, which indirectly modulates GSH synthesis (40). In myeloid cells, GSH metabolism regulation modulates BH3 analog sensitivity, influencing treatment efficacy in acute myeloid leukemia (41). However, the role of GSH metabolism within myeloid cells in PC remains unclarified. In PK cells, membrane-bound gemcitabine generated through NK cell-based adoptive cell transfer (ACT) exerts anti-tumor effects in PANC 1 cells by modulating GSH levels released during cancer cell lysis (42). Furthermore, a novel GSH isoform, S-geranylgeranyl-L-GSH, has been identified as a potent P2RY8 ligand with direct action on human B cells (43). In conclusion, aberrant GSH metabolism drives tumorigenesis by impacting multiple cellular components within the tumor microenvironment of PC, significantly influencing its chemotherapeutic drug sensitivity.

A prognostic correlation model based on GSH metabolism in PC was established, identifying six key model genes. Key genes were selected using random forest survival analysis, and the intersection of both analytical outcomes revealed four core genes: GSTA4, GSTA5, GPX2, and GPX3. The GSH S-transferase family (GSTs) encompasses multiple classes, each comprising various isozymes (44). As a pivotal member of this family, GSH S-transferase-α (GSTA) primarily facilitates GSH binding and detoxification within cells, while also playing essential roles in cell signaling, post-translational modification, and resistance to anticancer drugs (45, 46).

Evidence indicates that variations in GSTA gene expression can modulate the activity of GSTA isozymes, disrupting cellular redox homeostasis, promoting tumor cell proliferation, and linking GSTA gene mutations to an elevated tumor risk, particularly in cancers such as bladder and colorectal cancer (47, 48). Additionally, GST has been implicated in cancer drug resistance (49, 50). For instance, GST inhibitors demonstrate anti-proliferative effects on tumor cells, and accumulating studies suggest that GSTs are emerging as novel therapeutic targets (50, 51). Nonetheless, the precise role of GSTA in PC remains to be clarified. Validation through multiple PC cell lines indicated that GSTA4 expression was markedly upregulated in PC compared to normal pancreatic cells. Notably, this study is the first to identify GSTA4 as a prognostic protective factor capable of inhibiting the proliferation and migration of PC cells.

Despite the comprehensive analysis of the intricate roles of GSH metabolism-related genes in the tumor microenvironment using single-cell and transcriptome data from PC, and the successful *in vitro* validation of the key target GSTA4, the study has inherent limitations. The accuracy and stability of the GSH metabolism-related prognostic model necessitate further validation using external datasets. Additionally, the investigation of GSTA4’s functional phenotype in PC is at an early stage, and the mechanisms underlying GSTA4 downregulation remain unresolved.

## Conclusion

In conclusion, this study integrates bioinformatics analysis to elucidate the regulatory functions of GSH metabolism-related genes, utilizing single-cell and transcriptomic data from PC to construct a prognostic model incorporating six genes. As the first *in vitro* validation of GSTA4’s biological function in PC, the findings provide a potential foundation for developing novel therapeutic strategies against PC.

## Data Availability

The original contributions presented in the study are included in the article/**Supplementary Material**. Further inquiries can be directed to the corresponding authors.

## Supplementary material

Supplementary Figure 1 GSH metabolism signal scoring using Add, AUCell, and singscore algorithms, mapped onto UMAP landscapes of normal pancreatic tissue and pancreatic cancer tissue.

Supplementary Figure 2 GSH metabolism signal scoring using ssgsea, UCell, and Scoring algorithms, visualized on UMAP landscapes of normal pancreatic tissue and pancreatic cancer tissue.

Supplementary Figure 3 Ranking and cophenetic correlation coefficients derived from NMF rank determination.

Supplementary Figure 4 Immune checkpoint analysis based on NMF clustering.

Supplementary Figure 5 Drug sensitivity profiling associated with NMF clustering.

Supplementary Figure 6 Development of a prognostic signature based on GSH metabolism-related genes. **(A, B)** LASSO Cox regression analysis was employed to select optimal parameters. **(C)** Multivariate Cox regression analysis identified six GSH metabolism-related genes as key components of the prognostic model.

Supplementary Figure 7 Heatmap depicting the differential expression profiles of six prognostic model genes between high-risk and low-risk groups within the GSH metabolism-related prognostic model.
