# Supplementary figures and images for: Integrative single-cell RNA sequencing and bulk RNA sequencing reveals the characteristics of glutathione metabolism and protective role of GSTA4 gene in pancreatic cancer
Source: Front Immunol. 2025 May 1;16:1571431. doi: 10.3389/fimmu.2025.1571431 (PMC12078301; doi:10.3389/fimmu.2025.1571431)

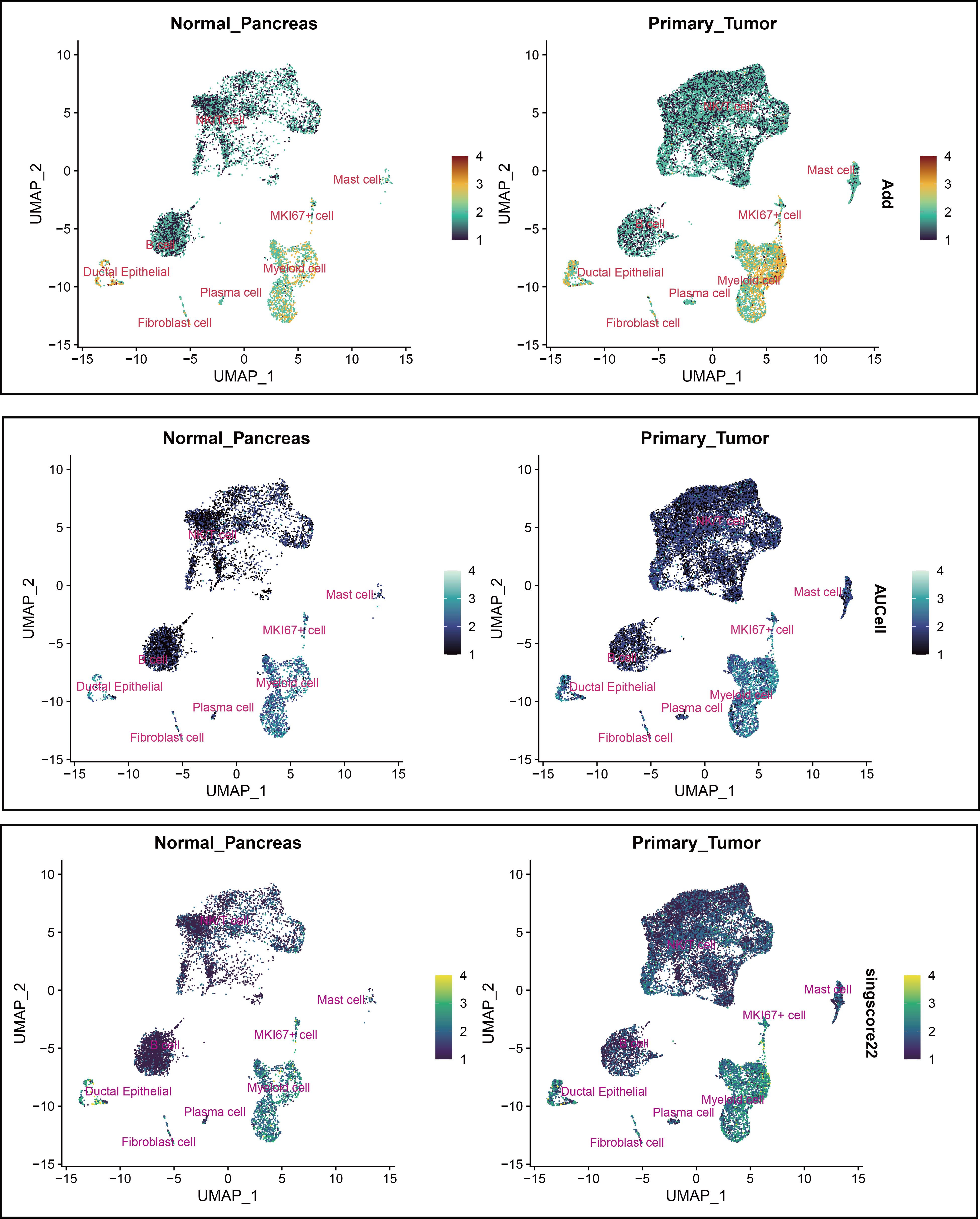

Supplement: Supplementary Figure 1 — GSH metabolism signal scoring using Add, AUCell, and singscore algorithms, mapped onto UMAP landscapes of normal pancreatic tissue and pancreatic cancer tissue. [file Image1.jpeg]

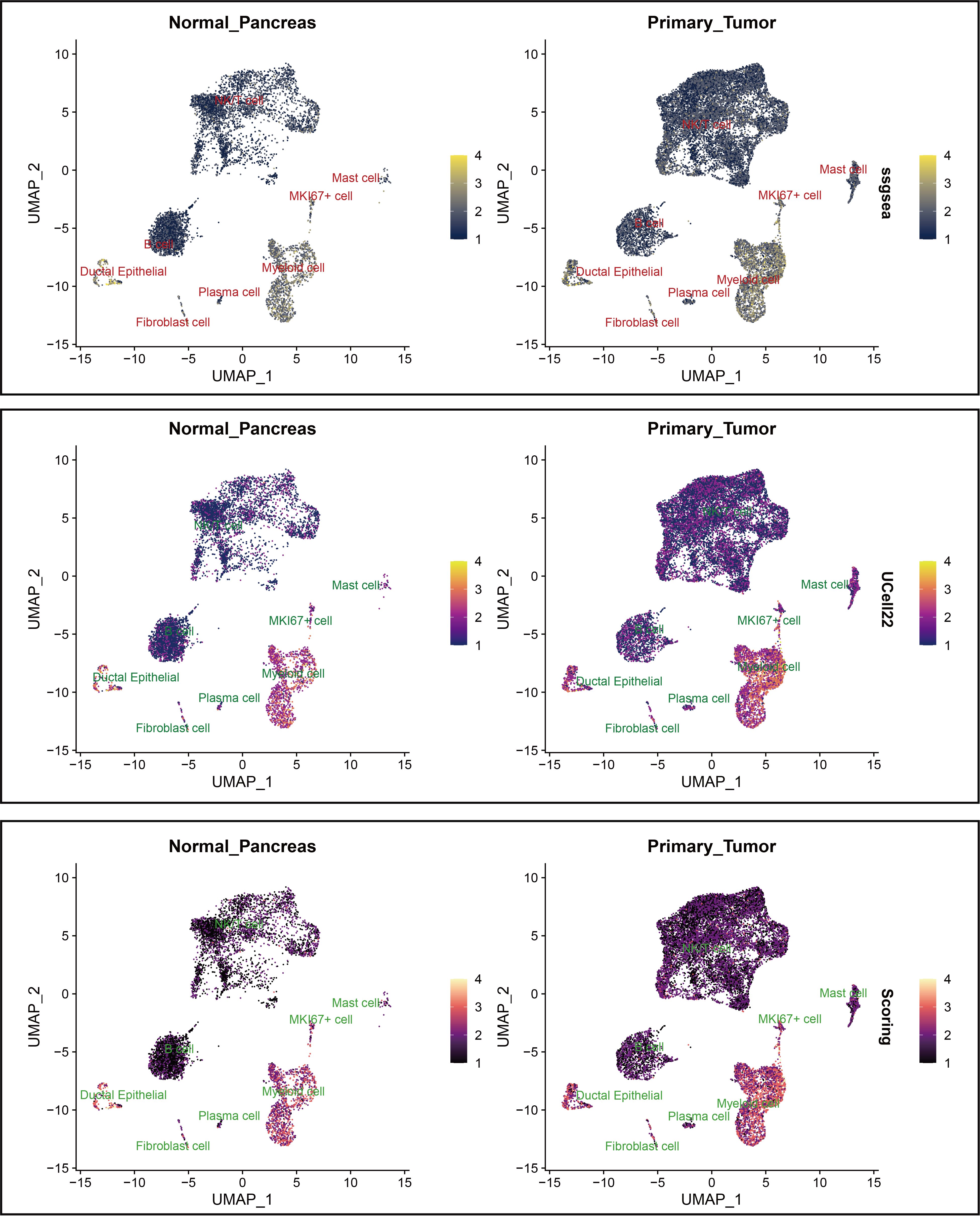

Supplement: Supplementary Figure 2 — GSH metabolism signal scoring using ssgsea, UCell, and Scoring algorithms, visualized on UMAP landscapes of normal pancreatic tissue and pancreatic cancer tissue. [file Image2.jpeg]

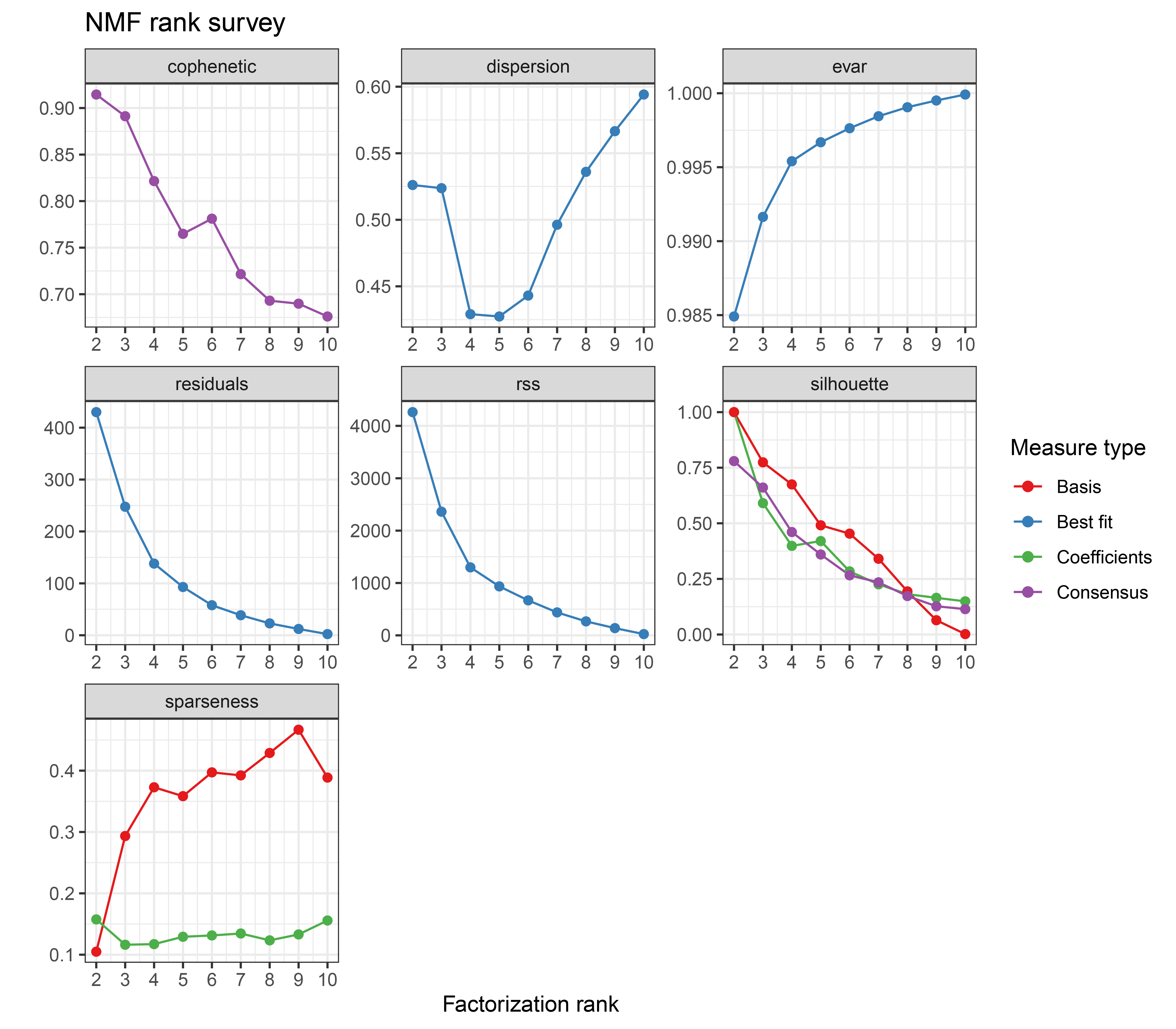

Supplement: Supplementary Figure 3 — Ranking and cophenetic correlation coefficients derived from NMF rank determination. [file Image3.jpeg]

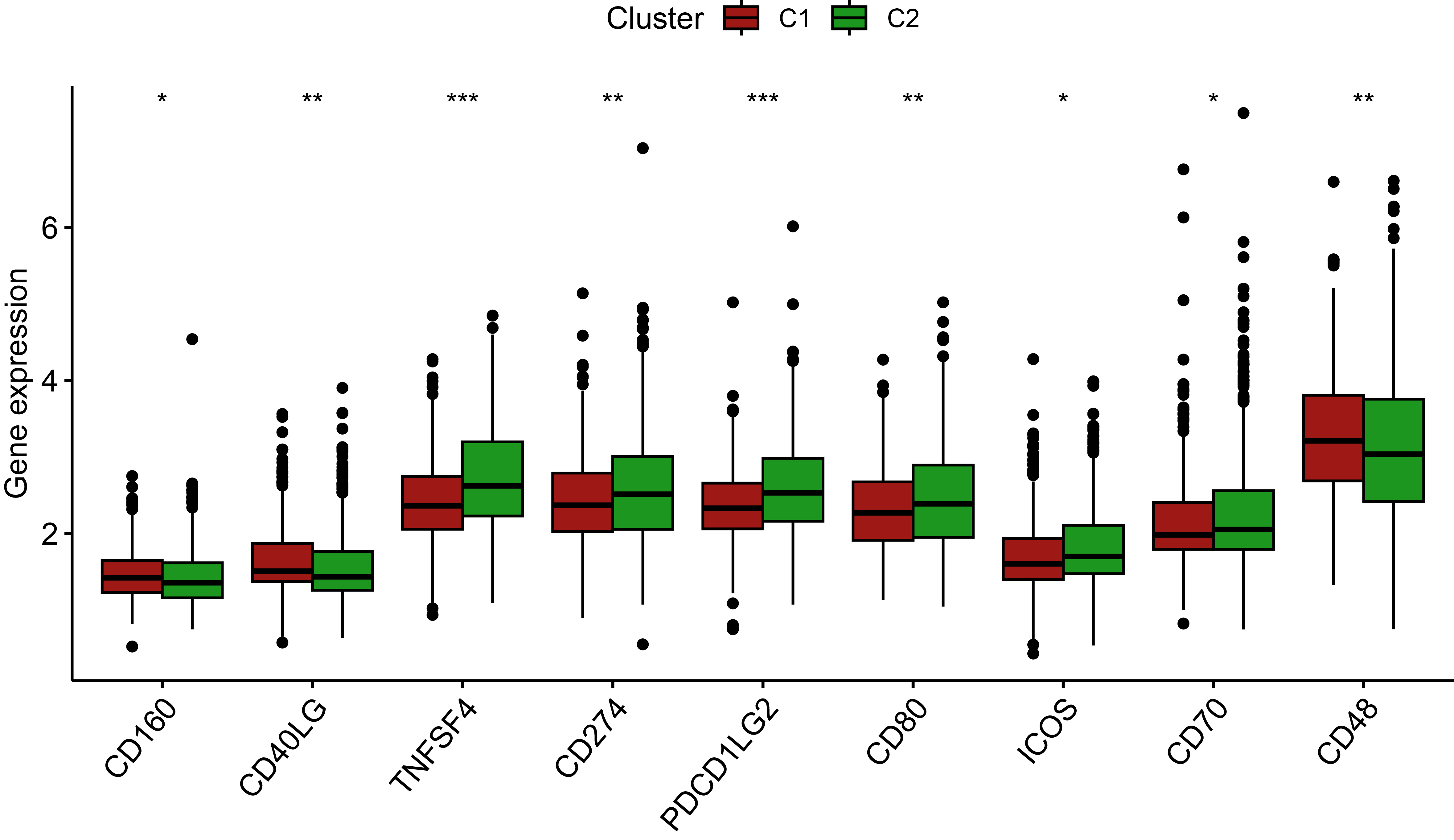

Supplement: Supplementary Figure 4 — Immune checkpoint analysis based on NMF clustering. [file Image4.jpeg]

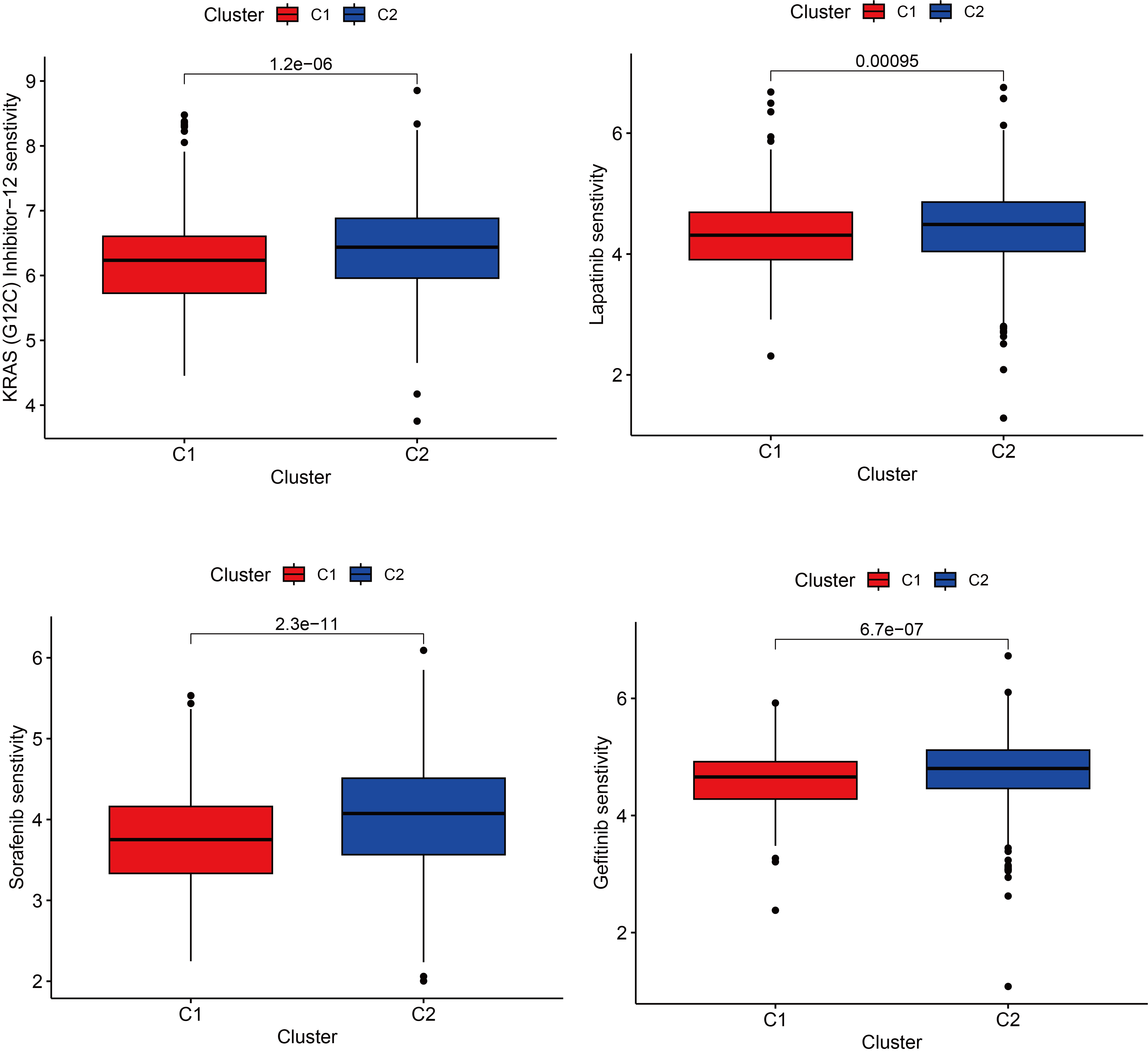

Supplement: Supplementary Figure 5 — Drug sensitivity profiling associated with NMF clustering. [file Image5.jpeg]

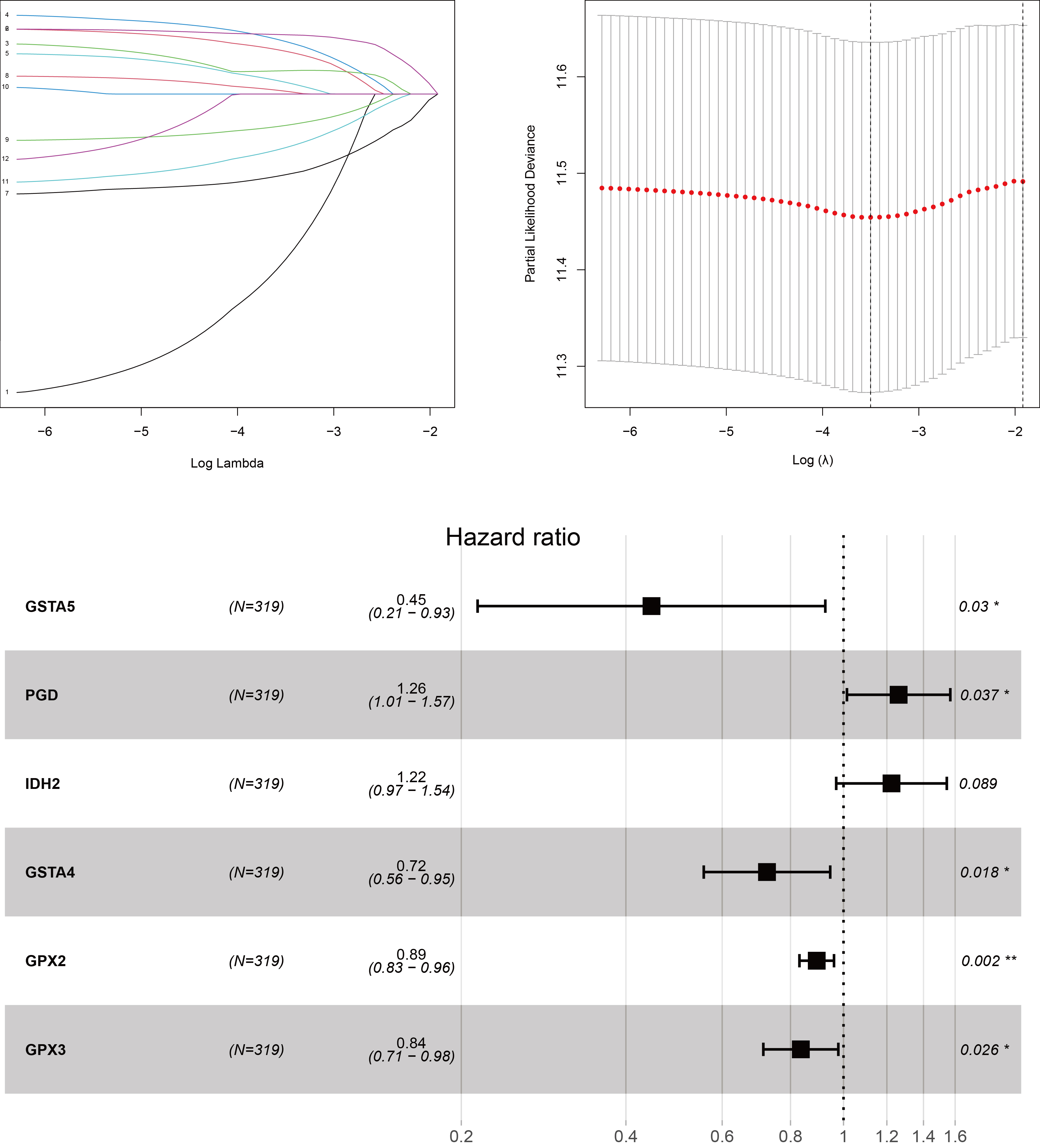

Supplement: Supplementary Figure 6 — Development of a prognostic signature based on GSH metabolism-related genes. (A, B) LASSO Cox regression analysis was employed to select optimal parameters. (C) Multivariate Cox regression analysis identified six GSH metabolism-related genes as key components of the prognostic model. [file Image6.jpeg]

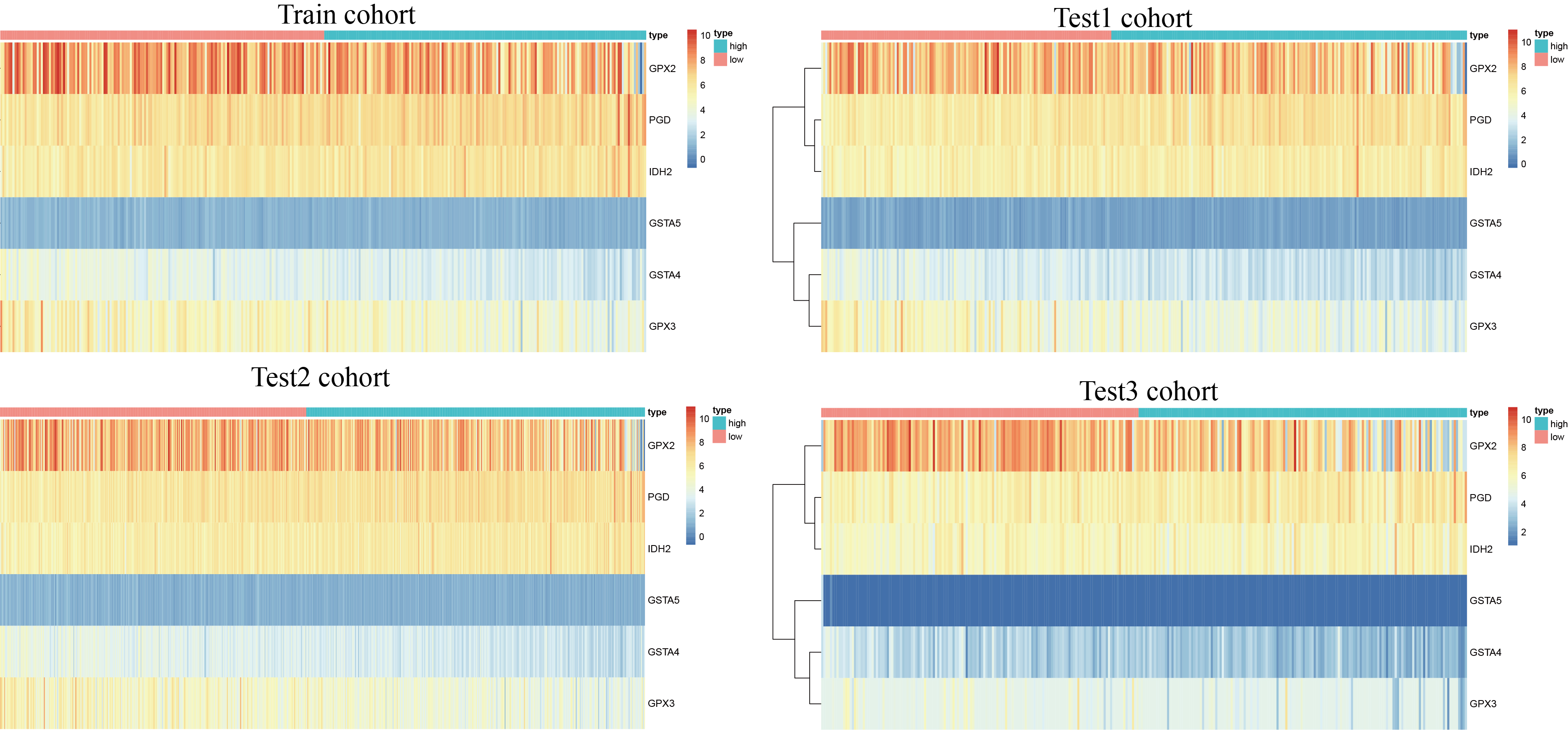

Supplement: Supplementary Figure 7 — Heatmap depicting the differential expression profiles of six prognostic model genes between high-risk and low-risk groups within the GSH metabolism-related prognostic model. [file Image7.jpeg]
